# Fermented Edible Insects for Promoting Food Security in Africa

**DOI:** 10.3390/insects11050283

**Published:** 2020-05-05

**Authors:** Yusuf Olamide Kewuyemi, Hema Kesa, Chiemela Enyinnaya Chinma, Oluwafemi Ayodeji Adebo

**Affiliations:** 1 School of Tourism and Hospitality, College of Business and Economics, University of Johannesburg, P. O. Box 524, Bunting Road Campus, Johannesburg, Gauteng, South Africa; hemak@uj.ac.za; 2 Department of Food Science and Technology, Federal University of Technology, P.M.B. 65, Minna, Niger State, Nigeria; chinmachiemela@futminna.edu.ng; 3 Department of Biotechnology and Food Technology, Faculty of Science, University of Johannesburg, P. O. Box 17011, Doornfontein Campus, Johannesburg, Gauteng, South Africa

**Keywords:** fermentation, edible insect, nutrition, healthy diets, sub-Saharan Africa, fermented foods, food security, food processing

## Abstract

Efforts to attain sustainable nutritional diets in sub-Saharan Africa (SSA) are still below par. The continent is envisaged to face more impending food crises. This review presents an overview of common edible insects in Africa, their nutritional composition, health benefits and utilization in connection with fermentation to enrich the inherent composition of insect-based products and offer foods related to existing and generally preferred culinary practice. Attempts to explore fermentation treatments involving insects showed fermentation affected secondary metabolites to induce antimicrobial, nutritional and therapeutic properties. Available value-added fermented edible insect products like paste, powder, sauces, and insect containing fermented foods have been developed with potential for more. Novel fermented edible insect-based products could effectively fit in the continent’s food mix and therefore mitigate ongoing food insecurity, as well as to balance nutrition with health risk concerns limiting edible insects’ product acceptability in SSA.

## 1. Introduction

Being multicellular eukaryotes representing the most abundant member of the phylum Arthropoda, insects were some of the earliest terrestrial life forms on Earth. The edible species provide important nutrients for many marginalized households in developing countries, although as of late they have also become increasingly popular in western industrialized societies. In comparison with conventional animal sources such as beef, chicken and pork, studies have documented that edible insects are better sources of dietary protein (31.06–71.04%), fat (7.00–50.50%), energy (313.44–625.82 kcal), as well as meeting the total recommended daily intakes of essential amino acids (310.90%) (Table 1). Variations in these values nonetheless exist relative to diversity among species [1,2]. However, Payne et al. [3] argue that insects are nutritionally not significantly different from conventional meats.

According to a 2017 estimate, 2111 species of edible insects have been recorded in the world [4]. Globally even today a significant number of people, mainly in Africa, Asia, South and Latin America still consume insects as food. An entomophagy survey carried out by Kelemu et al. [5] indicated that over 470 species of edible insects are unique to Africa, and they are majorly concentrated in Central Africa (Cameroon, Congo Republic–Brazzaville, Democratic Republic of the Congo and the Central African Republic), Southern Africa (South Africa, Zambia and Zimbabwe) with other notable countries being Uganda and Nigeria. Commonalities exist within regions of the continent and these insects are seasonally available. Frequently utilized types are caterpillars (*Cirina forda*), mopane worms (*Gonimbrasia belina*), termites (*Macrotermes* spp.), crickets (*Brachytrupes membranaceus*, *Gryllus* spp.), palm weevils (*Rhynchophorus phoenicis*), grasshoppers (*Ruspolia differens*), locusts (*Nomadacris septemfasciata*), stinkbugs (*Encosternum delegorguei*), beetles (*Oryctes* spp.), ants (*Carebara* spp.) and bees (*Apis* spp.) [5]. Based on metamorphic life stage (eggs, larva/nymph, pupae or adult), edible insects have been consumed after minor processing or ingested after preparation by parboiling, sun drying, curing, smoking, roasting, stewing, frying or combination of these processes thereof and afterward, they are subsequently consumed either solely, as a snack or in soups if desired [6,7]. Thus, consumption patterns are just about the same as for warm-blooded animals and seafood.

Food security in its entirety refers to the provision of safe, nutritious and sufficient foods by means of unrestricted availability and access to the populace and it was suggested as early as 1975 by Meyer-Rochow [8] that insects could help to ease the problem of global food shortages. Most food preparation in Africa is usually based on community/generational preferences and eating quantity rather than the overall quality needed to balance healthy living. The resultant significant effect manifests itself in the form of mal- and undernourishment which has been on the increase. A recent estimate by FAO on the nutritional status of SSA populace indicates 22.8% level of undernourishment, 239.1 million undernourished individuals and 605.8 million number of moderately or severely food-deprived individuals [9]. This is further compounded by the recent rise in food prices of commonly traded food commodities [10]. In line with the UN’s sustainable development goal of zero hunger, intensified efforts are needed towards improving food value by cost effectively processing edible materials in such a way that the nutritional components are enhanced to meet the daily recommended dietary allowances and providing foods that can confer health benefits.

Although substantial progress has been made with improvements in food processing techniques and significant contributions in conversion of farm produce to edible food products, it is not surprising that time-honored old fermentation technologies are still frequently adopted, either singly or applied in tandem with other techniques to obtain novel food products. Fermented food products have appealing characteristics, enhanced nutritional bioavailability, health-promoting constituents as well as improved organoleptic characteristics. Fermentation as a process stimulates the production of bioactive compounds which may be initially deficient or available in minute quantities in the unprocessed substrate [11]. Fermented foods have been mostly explored from plant-based substrates (such as grains, fruit and vegetables), dairy and common meat sources and fish, with less interest on edible insects.

Using a narrative (semi-systematic) approach, the review appraised available studies and provided an overview of common edible insects in Africa, their nutritional composition, health benefits as well as potential application of fermentation to produce edible insect foods of varying kinds that can consequently enrich the food basket of the continent and contribute to food security. Information was sought from major scientific databases using the relevant keywords, collated and summarized. The review also discussed the acceptability of insect-based products and concluded with further areas and efforts needed regarding fermented edible insects and food security in the continent.

## 2. Common Edible Food Insects in Africa

**Figure 1:**
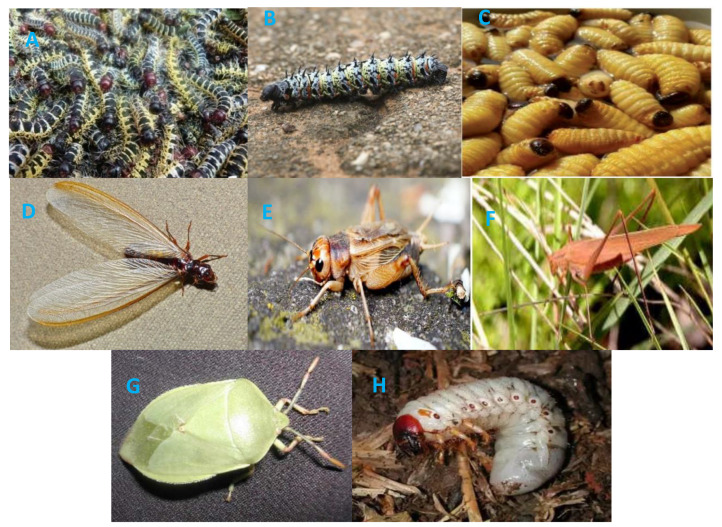
Common African edible insects: (**A**) *Cirina forda* (moth caterpillar) (**B**) *Gonimbrasia belina* (mopane ‘worm’ = caterpillar) (**C**) *Rhynchophorus phoenicis* (palm weevil grubs) (**D**) *Macrotermes* spp. (winged adult termite) (**E**) *Gryllus assimilis* (adult cricket) (**F**) *Ruspolia differens* (adult grasshopper) (**G**) *Encosternum delegorguei* (adult stink-bug) (**H**) *Oryctes rhinoceros* (palm beetle grub) (Adapted from [20,29,30,31,32,33,34,35]).

Most available edible insect species belong to the order Lepidoptera, Coleoptera and Isoptera. Others include Orthoptera and Hemiptera, which are also widespread in the continent (Table 1). They are classified in suborders existing in super-families and excessive species that aid environmental processes. Food insect species are morphologically characterized to have a three-part body consisting of the head, thorax and abdomen; chitinous exoskeleton, six jointed legs, a pair of antennae and compound eyes (Figure 1). Species numbers and insect densities are higher in tropical regions, where some insects are periodically super-abundant; hence, they were salted to enhance preservation during the first century BC [24]. This shows that entomophagy has long been practiced by humans with distinct preference for some insect species as a form of delicacy and strong ties with ethnicity. This however does not necessarily seem to be the case as some ethnic groups see the consumption of insects or certain types of insects as a taboo [25]. For instance, only 11.8% of 203 Ethiopian respondents versus 45.6% of Koreans reported that they were willing to serve a meal containing insects [26]. Furthermore, Fasoranti and Ajiboye [27] illustrated blacksmiths of the Ire lineage, Yoruba tribe in Nigeria avoid eating crickets because they worshipped the Iron God ‘Ogun’ which does not accept live offerings that have no blood. Similar differences in preference of food insects exist among cultural diversities in the world and are well known [28].

## 3. Nutritional Composition and Health Benefits of Edible Insects

The nutritional and other edible food constituents contribute greatly to sustaining healthy life. Studies have dealt with the nutritional composition of edible insects in terms of their quality protein, fat and essential micronutrients with levels close to those reported in other food sources [2,28]. According to Bruinsma [36], repeating an earlier call by Meyer-Rochow [8], edible insects are considered the main solution to the challenges of meeting the growing global demand for animal protein that is generally sought after for its high nutritional value. The protein content of edible insects on dry basis ranged from 31.06 to 71.04% (Table 1).

The energy value of edible insects depends on the available crude fat and proportion of fat composition which varies from saturated, mono- to poly-unsaturated fatty acids [2]. Some nutritionally important fatty acids have been reported for mealworms, bush-crickets and grasshoppers with such studies indicating the appreciable levels of these lipid fractions with some linked to health-promoting effects such as regulation of the nervous system and protection against the risks of cardiovascular diseases [37,38,39,40,41]. Carbohydrates are available in form of indigestible chitin (dietary fiber) representing the chief constituents of insect exoskeletons [42]. Lower digestibility of cricket protein has been reported and was associated with the presence of chitin [13], a component which could be decomposed by food processing technique such as fermentation to liberate nutritional substances.

The variation in the biochemical composition of edible insect species is primarily due to their different raising habitats and diets as well as metamorphic life stage and mode of food preparation [43]. As such, specific or comparative investigations on common edible insect species have reflected this variation (Table 1). Most often, dried food insect larvae/grubs/nymph or at adult stage were evaluated for nutritional compositions. These edible insects contain sufficient amounts of protein, fat and minerals, as well as considerable levels of fiber and carbohydrate (Table 1). *Cirina forda* and *Gryllus assimilis* were demonstrated to contain the full profile of essential amino acids with average low levels of methionine and a high amount of leucine. Notably across some of these edible insect species is the relatively high level of macro minerals such as calcium, magnesium, potassium, phosphorus, sodium and average quantities of iron and zinc. The minerals are not only abundant but also readily available and due to high contents of iron and zinc, could assist in alleviating mineral deficiencies in the continent [44]. Likewise, are measurable levels of vitamins and acceptable levels of anti-nutritional components (Table 1). While anti-nutritional components are primarily responsible for inhibiting essential nutrient bio-accessibility, they are considered as health promoting substances at low levels [45]. Popova and Mihaylova [46] reported body glucose and triacylglycerols reduction effects by these compounds. Interestingly, anti-nutrient quantities estimated in edible insect species are similar to the permissible limits of staple plant crops and as such possess little risk and are of no concern [12].

The enormous potential benefits of edible insects have been demonstrated to far exceed the nutritional significance to functional and health prospects partly due to the presence of microscopic internal organs that generates metabolic products, which in turn elicit pharmacologically vital compounds, such as peptides, dietary antioxidants, etc. [47,48]. Polyphenols are important bioactive compounds that possess antioxidant properties which inhibit the risk of cancer, cardiovascular, neurodegenerative and metabolic diseases by limiting oxidative stress and inflammatory reactions, and subsequently enhance immune antioxidant defense mechanism [49]. Studies investigating polyphenol levels in edible insects have indicated levels between 0.37–500 mg GAE/100 g [50,51,52,53,54], with these polyphenols contributing to health promoting properties. *Apis mellifera* brood powder has been demonstrated to possess high antioxidant activity and beneficial monounsaturated and saturated fatty acids linked with reduced blood lipids [52]. To further explore the connection between edible insect composition and human gut microbiota to potentially induce health benefits, in vitro fecal models were used to simulate metabolic events of digested *Tenebrio molitor* flour and the result showed an increase in health promoting bacterial groups and the formation of vital end products, namely short- and branched-chain fatty acids [55]. Similarly, hydrolysates from grounded raw *T. molitor* and *Gryllodes sigillatus* significantly stimulated the growth of human skin fibroblasts [56]. In addition, bioactive edible insect extracts of *Acheta domesticus* and *T. molitor* have been characterized to be principally rich in amino acids, carbohydrates, free fatty acids, hydrocarbons organic acids and sterols, and as well assayed to demonstrate duo antioxidant activity and inhibitory effect of pancreatic lipase enzyme, a potential mechanism which could confer therapeutic effects [54].

According to Meyer-Rochow [48] and Hwang et al. [57] insects are customarily used in oriental medicines, with insect extracts recently been investigated for medical assays [47,57]. The protective effects of *Gryllus bimaculatus* extracts against acute alcoholic liver damage in mice was more potent to gut-derived inflammatory responses (alcohol-induced steatosis and apoptotic) in the liver and intestinal permeability to bacteria endotoxin in the small intestine than other known therapeutic agents for the disease treatment [57]. Similar insect species have also been documented to potentiate significant anti-aging and anti-inflammatory effects (Table 2) in rat models [47,58]. In vivo assay of a related genus, *Gryllus assimilis* revealed that a cricket diet lowered serum low density lipoprotein cholesterol concentration, suggesting a potential decrease in the risk of cardiovascular diseases [13]. This effect has been attributed to the role of chitin in reducing serum cholesterol and aiding hemostasis processes in the human body [59]. Further increases in the bioavailability of inherent health-benefitting constituents and nutrients of edible insects could be aided by controlled fermentation, a process described in the next section of this review.

## 4. Fermentation of Edible Insect

The cultural richness of African inhabitants maybe traced to their simple practices of converting their farm produce to edible and preservable forms essential for sustaining their existence. A commonly used technique is fermentation, a centuries old practice aided by uncontrolled microbes (natural or spontaneous and use of natural starter, i.e., backslopping) or controlled microflora (starter culture) which subsequently leads to desirable food properties like distinct organoleptic characteristics, better palatability and extended shelf life [60]. Simultaneously, the process is also accompanied with the improvement of biochemical properties and depletion/elimination of toxic components of the inherent raw material [61]. Fermentation processes also stimulate the production of bioactive compounds which maybe initially lacking or be present in small quantities in the unprocessed substrate [11].

Fermentation has been adopted as a technique for improving the shelf life and microbial safety of edible insects with studies indicating the role of fermentation in suppressing the growth of pathogenic microorganisms [62,63]. Addition of a lactic acid bacteria (LAB) starter during the fermentation of *Tenebrio molitor* was observed to have inhibited undesirable microorganisms, particularly sulfite reducing clostridia [63]. Similar observation was equally noted in the study of De Smet et al. [41], with a prevention in growth of unwanted microorganisms reported. Through microbial activity during fermentation organisms, a reduction in pH is expected and this reduced level provides a barrier against most foodborne pathogens. Although several microorganisms (bacteria, yeast and fungi) are generally involved in the food fermentation process [45,60,61,64], however LABs and yeast are the most notable ones and have been documented for their role to confer potential health benefits in fermented edible insects [41].

Nonetheless, differences exist between the fermentation processes of plant and animal-based material. The former is genetically made up of carbohydrate and sugar substances which are essential nutrient for catalyzing the action of fermenting microbes to enable a suitable acidification, whereas the latter contain less of these substances (excluding milk substrate) but are rich in proteins that are susceptible to the actions of various microorganisms. Edible insects are therefore pretreated (drying, blanching, boiling, roasting, crushing/size reduction, salting or smoking) before initiating fermentation [62,63,65]. These preprocesses are essential to remove the protective barrier (exoskeleton) and for exposing essential nutrients needed for microbial activities during fermentation. The resulting pH of subsequent fermented products may at times not reach a value necessary to prevent the survival/growth of pathogenic bacteria. In such cases, use of other preprocessing techniques, starter cultures and/or the use of preservatives may be added to the substrate to inhibit unwanted microbiota.

Regardless of the massive exploitation of fermentation processes for other food sources, relatively few studies have utilized fermentation for processing edible insects into food intermediates or incorporated them in common fermented foods (Table 3 and Table 4). Promising laboratory-scale preparation of novel fermented insect-based foods involve adequate pretreatment of edible insect larvae, further ground to completely no particles to obtain substrate that could be readily metabolized by varying starter cultures and incorporation of preservative to restrain interference of background microflora [41]. Similarly, powdered edible insects free of particles could also form part of composite flour with cereal for fermentation by making repetitive acidification of the flour (back-slopping) and part-inclusion of the natural starter for the fermentation process without addition of preservatives. Such have also been shown to suppress the activity of unwanted microbes [62].

As discussed in Section 4, the prospect of fermentation to improve the bioavailability of elementary nutritious and health-promoting components in fermented edible insects is feasible. Available studies have employed starter cultures (mainly bacteria and fungi culture) in either submerged or solid-state fermentation at varying conditions to enable bio-modification of inherent components of edible insects. Among the few experimental studies, solid state fermentation of dried *Bombyx mori* larvae using *Aspergillus kawachii* as starter culture was reported to show higher fatty acid and lower amino acid and mineral contents [66]. Ethanol extracts from the resultant sample was therapeutically demonstrated to induce improved cancer inhibitory effects in human hepatocellular carcinoma cells through modification of secondary metabolites. The filamentous fungus *Aspergillus oryzae* was similarly assayed for submerged fermentation using *Galleria mellonella* larvae and *Locusta migratoria* as substrate and showed high levels of glutamate and aspartate [65]. Likewise, imitation of soy sauce fermentation for *Tenebrio molitor* larvae liquid fermented seasoning using *A. oryzae* and *Bacillus licheniformis* revealed that the raw insect sauce showed an optimal free amino acid content as well as the production of compounds contributing to desirable flavor of the product [67]. On the other hand, fermented pastes of *T. molitor* larvae brought about by the action of lactic acid bacteria culture with added preservatives [2.8% NaCl (*w*/*w*), 0.75% D(+)-glucose (*w*/*w*) and 0.015% NaNO_2_(*w*/*w*)] gave rapid acidification and a suitable condition that inhibited the presence of bacterial endospores and sulfite-reducing *Clostridia* [41,63].

## 5. Incorporation of Edible Insects into Generally Acceptable Fermented Foods

To date, baked foods represent the most important nutrient vehicle due to their wide acceptance. Thus, recent studies are utilizing such product as a means of incorporating edible insects to prepare acceptable foods. A handful of trials have utilized insects suitable for the production of baked foods (e.g., buns, biscuits, cookies) in Africa [1,75,76]. Fermented edible insects have also been well processed into intermediate food products such as flour, paste and sauces through back-slopping and controlled fermentation (Table 3 and Table 4).

In a study on increasing the nutritional quality of bread through the incorporation of innovative ingredients, substitution of cricket powder prior to fermentation and subsequent baking to obtain bread resulted in a higher nutritional profile in terms of fatty acid composition, protein content and occurrence of essential amino acids [68]. The authors reported higher titratable acidity after fermentation in doughs and breads containing cricket powder, attributing this to higher ash content of the cricket powder which may have had an influence on dough buffering capacity. Compared with other substituted ratios, bread enriched with 10% cricket powder reportedly showed a discrete acceptability by untrained panelists, with the study suggesting that foods where insects are not directly visible could be more successfully marketed [68]. Even though probands blindfolded and unable to smell had trouble to identify insects by taste alone [77]. Similar observations were also demonstrated by González et al. [69] using other edible species. Furthermore, composite-gluten free breads mainly containing 10% and 20% inclusion of cricket powder, rice flour, corn starch and hydrocolloids (carboxymethyl cellulose and xanthan gum) have been demonstrated to show significantly higher protein (40% and 100%, respectively) and lipid content compared with the control sample. Technological assessment of the insect based-bread with no added oil gave desirable reduction in hardness and chewiness and other acceptable technological properties [70]. As such, bread containing insect with no added oil or otherwise with defatted insect could enhance good quality baked products.

Insects have also been used in the preparation of sauces with resulting production eliciting superior sensory qualities, with probability for wider use and utilization on a commercial basis [65]. In another study to enhance nutritional functionality and product quality of insect containing fermented foods, 0.5% bioactive peptides obtained from degreased dried silkworm pupae enhanced amino acid content, acidification and ACE inhibitory activity and gave better firmness and consistency yogurt. However, inclusion levels greater than 0.3% were attributed to unusual yogurt flavor [73]. In essence, the highlighted studies suggest that edible insect species have been explored for novel food development with potential to utilize the highlighted ones and other edible insect species for the sustainable production of acceptable food products. However, there is paucity of information in the literature on quality attributes of fermented edible insect proteins for the enrichment of cereal-based foods considering the health benefits associated with the consumption of fermented proteins.

Microbial quality of edible insects and their products therefore is of significant concern as documented in the European Food Safety Authority (EFSA) scientific opinion document and other published articles [68,78,79]. This suggests potential safety issues for consumers which necessitate adequate pretreatment of these edible insects. Fermentation has equally been adopted as one of such pretreatments as demonstrated in the study of Klunder et al. [62] which reported antimicrobial effects of fermenting composite meal of sorghum flour substituted with 10% or 20% powdered roasted mealworm larvae, with the resulting product effectively inactivating pathogenicity of Enterobacteriaceae while proliferation of spore forming bacteria was controlled in the acidic medium. A similar edible species was blanched and also tried for fermentation using combined inoculation of commercial meat starter culture and addition of preservative agent (previously used for meat fermentation) to larvae paste resulting in rapid acidification and inhibition of undesirable microbial flora that was unattainable in the spontaneously fermented portion despite the addition of preservatives [63]. These studies suggest fermentation as a promising strategy with potential to control unwanted microbial growth in insect paste.

## 6. Consumer Acceptance of Edible Insects

There is a growing market for edible insects with a recent forecast suggesting that the edible insect market will be worth approximately 8 billion US dollars by 2030 [80]. While this is interesting to note, there are still issues of neophobia (unwillingness to consume novel products) and food safety challenges with insects [81]. In addition, are issues of unfamiliarity, socio-cultural influence, prejudice, flavor, disgust and emotional concerns with consumers [82,83,84]. Moreover, whether edible or not, insects are generally perceived as annoying pests or dangerous vectors of disease. Such mindset influences food choices with sight possibly playing a role in this. This is substantiated by findings of a blindfolded sensory taste experiments in which participants had difficulty identifying edible insect products [77]. Although consumers cannot necessarily be blindfolded when purchasing food products, processing methods such as fermentation singly and in combination with other techniques can help promote the consumption of edible insects as subsequent products will be in an unrecognizable form. Innovative processing technologies could also be applied, as demonstrated in a study where three-dimensional (3D) printed snacks containing edible insect were produced [85]. Such products could be more appealing leading to better acceptability.

## 7. Future Prospects and Conclusions

The growing human population in the continent which is expected to be over 8 billion in 2025 [86] would increase the demand for food protein sources for human consumption. The application of fermentation processes in converting edible insects into nutritious and healthy foods could in part contribute significantly towards achieving a food-secure nation and thus attaining the UN’s millennium development goal of zero hunger in Africa. The ubiquitous nature of edible insect in SSA could fill the gap of healthy fermented foods required for a sustainable healthy lifestyle. In the Western world, varying innovative fermented insect-based foods such as extract, paste, powder sauces and insects containing fermented foods (composite bread) have been prepared and demonstrated promising prospects in terms of nutritious and healthy diets. However, of huge concern is ensuring optimum microbial stability and further research to establish the suitability of incorporating the edible insect intermediates into other food mixtures to enable different modes of utilization and to provide options for highly demanding consumers. Consuming edible insect-based foods could be explored as a viable way of addressing food insecurity in Africa as these insects are of high nutritional quality, readily available, cheap/affordable and their propagation is associated with negligible environmental footprints. While consumer issues may be challenging, this can be surmounted by incorporating fermented edible insects into snacks/products that would mask their appearance and increase acceptance of this product. Furthermore, to ensure the food security prospect of edible insects, much is still needed to be done in the areas of agricultural productivity. To successfully balance the need to achieve food security with the risk of depleting the edible insect resource and to address the problem of the ‘disgust factor’ still requires a lot of work as Gahukar [87] so aptly has pointed out in his review.

## Figures and Tables

**Table 1:** Nutritional composition of frequently investigated edible insect species in Africa in comparison with common sources of animal protein.

Nutritional Components	*Cirina Forda* (Moth Caterpillar: Lepidoptera)	*Encosternum Delegorguei* (Stink-Bug: Hemiptera)	*Gonimbrasia Belina* (mopane Worm/Caterpillar: Lepidoptera)	*Gryllus Assimilis* (Cricket: Orthoptera)	*Macrotermes Bellicosus* (Termite: Isoptera)	*Oryctes Rhinoceros* (Palm Beetle: Coleoptera)	*Rhynchophorus Phoenicis* (Palm Weevils: Coleoptera)	*Ruspolia Differens* (Grasshopper: Orthoptera)	Beef	Chicken	Pork	Recommended Daily Intakes (mg/kg Body Mass of Adult/Day)
Proximate composition (%)												
Protein	69.31	35.20	55.41	71.04	31.06	52.00	45.44	44.30	22.30	22.80	22.80	
Fat	10.64	50.50	16.37	7.00	39.82	10.84	41.84	46.20	1.80	0.90	1.20	
Energy (kcal/100 g)	406.84	625.82	401.61	397.00	496.50	313.44	584.68	-	115.92	104.92	112.09	
Fiber	5.97	-	-	8.28	1.69	17.94	7.55	4.90	-	-	-	
Dry matter	-	95.10	-	-	-	-	-	-	-	-	-	
Carbohydrate	8.48	7.63	8.16	12.46	3.48	1.97	6.59	-	-	-	-	
Moisture	4.02	4.90	-	3.50	22.79	5.42	26.00	-	75.00	75.00	75.10	
Ash	6.16	1.70	8.26	6.00	1.18	11.83	5.71	2.60	1.20	1.20	1.00	
Essential amino acid (g/100 g)												
Histidine	2.08	-	-	2.52	29.60	-	1.10	-	2.90	4.40	3.20	10
Isoleucine	3.68	0.83	-	3.36	49.00	-	2.40	-	5.10	4.20	4.90	20
Leucine	5.91	1.05	-	6.62	62.10	-	4.70	-	8.40	6.90	7.50	39
Lysine	4.59	0.85	-	5.29	40.80	-	4.20	-	8.40	7.80	7.90	30
Methionine	0.62	0.40	-	2.29	30.30	-	2.10	-	2.30	2.10	2.50	10
Phenylalanine	4.64	0.81	-	3.37	40.50	-	6.50	-	4.00	2.50	4.10	25
Threonine	5.19	0.82	-	3.09	17.10	-	2.90	-	4.00	3.70	5.10	15
Tryptophan	1.84	0.16	-	2.53	-	-	-	-	-	-	-	4
Valine	5.10	1.32	-	4.63	41.50	-	4.10	-	5.70	4.60	5.00	26
Total EAA	33.65	6.24	-	33.70	310.90	-	28.00	-	40.80	36.20	40.20	179
Non-essential amino acid (g/100 g)												
Alanine	3.80	-	-	6.23	48.60	-	7.60	-	-	-	-	
Arginine	5.35	-	-	4.14	39.40	-	2.40	-	6.60	6.40	6.40	
Aspartic acid	7.82	-	-	8.25	69.60	-	-	-	-	-	-	
Cystine	0.66	-	-	1.14	17.30	-	2.50	-	-	-	-	
Glutamic acid	8.94	-	-	10.60	179.20	-	-	-	-	-	-	
Glycine	5.29	-	-	4.03	53.80	-	4.80	-	-	-	-	
Proline	3.26	-	-	5.09	19.10	-	10.20	-	-	-	-	
Serine	3.80	-	-	3.80	38.70	-	3.30	-	-	-	-	
Tyrosine	3.81	-	-	4.23	25.70	-	6.00	-	3.20	3.50	3.00	
Total NEAA	42.73	-	-	47.51	491.40	-	36.80	-	9.80	9.90	9.40	
Mineral composition (mg/100 g)												
Calcium	22.43	91.00	16.00	0.09	18.40	12.54	59.65	24.50	-	-	-	1300.00
Chlorine	-	85.40	-	-	-	-	-	-	-	-	-	-
Cadmium	-	-	-	-	8.40	-	-	-	-	-	-	-
Chromium	0.01	-	-	0.01	3.70	-	-		-	-	-	-
Copper	0.03	4.40	-	0.02	89.95	0.01	-	0.47	-	-	-	1.10
Iron	3.61	20.20	12.70	0.15	42.38	8.57	20.03	13.01	-	-	-	33.00
Magnesium	40.88	109.00	4.10	8.92	185.50	10.13	101.62	33.06	-	-	-	240.00
Manganese	0.64	0.80	-	0.00	119.13	0.39	2.92	2.46	-	-	-	2.20
Phosphorus	108.57	575.00	14.70	-	385.15	75.57	417.60	121.00	-	-	-	700.00
Potassium	223.34	275.00	35.20	367.13	229.00	25.44	546.78	259.70	-	-	-	4700.00
Selenium	-	0.20	-	-	0.01	-	-	-	-	-	-	0.03
Sodium	25.79	55.30	33.30	0.42	396.10	21.37	56.18	229.70	-	-	-	1500.00
Sulfate	-	66.70	-	-	-	-	-	-	-	-	-	-
Zinc	1.99	46.00	1.90	0.24	7.65	10.10	10.15	12.38	-	-	-	8.50
Vitamins (mg/100 g)												
Vitamin A	0.24	0.23	-	2.90	11.37	-	-	2.75	-	-	-	
Vitamin B_1_	-	0.63	-	-	0.87	-	-	-	-	-	-	
Vitamin B_2_	-	0.86	-	0.23	0.32	-	-	1.36	-	-	-	
Vitamin B_3_	-	-	-	-	1.59	-	-	2.36	-	-	-	
Vitamin B_6_	-	-	-	-	1.09	-	-	0.16	-	-	-	
Vitamin B_9_	-	-	-	-	-	-	-	0.92	-	-	-	
Vitamin B_12_ (g/100 g)	0.00	-	-	0.01	-	-	-	-	-	-	-	
Vitamin C	1.01	-	-	1.01	3.58	-	-	0.14	-	-	-	
Vitamin D	-	-	-	-	2.22	-	-	-	-	-	-	
Vitamin E (g/100 g)	0.36	2.17	-	0.33	3.60	-	-	0.15	-	-	-	
Vitamin K (g/100 g)	0.02	-	-	0.04	-	-	-	-	-	-	-	
Anti-nutritional composition (mg/100 g)												
Alkaloids	8.33	-	-	-	-	190.00	15.76	-	-	-	-	
Cyanogenic glycosides/hydrogen cyanide	11.75	-	-	3.76	-	-	-	-	-	-	-	
Flavonoids	3.44	-	-	-	-	240.00	5.39	-	-	-	-	
Oxalate	13.50	-	-	20.93	-	1.09	9.74	-	-	-	-	
Phytate	12.77	-	-	0.10	-	16.10	19.39	-	-	-	-	
Phytin phosphorus	-	-	-	-	-	4.53	-	-	-	-	-	
Polyphenol	0.37	-	-	-	-	-	0.56	-	-	-	-	
Tannins	0.51	-	-	0.49	-	0.64	0.61	-	-	-	-	
Saponins (g/100 g)	1.21	-	-	1.00	-	1.34	0.02	-	-	-	-	
	[12,13,14]	[6]	[15]	[12,13]	[16,17]	[18]	[14,19]	[20]	[21]	[22]	[22]	[23]

Average values are indicated in cases of varying reports on specific edible insect species. “-” = Not reported, EAA = essential amino acids, NEAA = non-essential amino acids. Values are expressed in dry matter.

**Table 2:** Summary of the reported health benefits of edible insects.

Insect Species	Administered Form	Health Benefits	References
*Apis mellifera*	Powder	High antioxidant activity and beneficial fatty acids linked with reduced blood lipids.	Haber et al. [52]
*Acheta domesticus and Tenebrio molitor*	Extract	Demonstrated antioxidant activity and inhibitory effect of pancreatic lipase enzyme.	Navarro del Hierro et al. [54]
*Gryllus assimilis*	Powder	Lowered serum low density lipoprotein cholesterol concentration.	Oibiokpa et al. [13]
*Gryllus bimaculatus*	Extracts	Potent against gut-derived inflammatory responses in the liver and intestinal permeability to bacteria endotoxin in the small intestine.	Hwang et al. [55]
*Gryllus bimaculatus*	Extract	Anti-aging and anti-inflammatory effects.	Ahn et al. [47,58]
*Tenebrio molitor*	Flour	Increase in health-promoting bacterial groups and formation of vital end products.	de Carvalho et al. [55]
*Tenebrio molitor* and *Gryllodes sigillatus*	Hydrolysates	Stimulated the growth of human skin fibroblasts.	Zielińska et al. [56]

**Table 3:** Fermentation treatment on edible insect species and resultant products.

Insect Species	Starter Culture	Fermentation Process and Condition	Food Products	Findings	References
*Galleria mellonella* (wax moth larvae), *Locusta migratoria* (grasshoppers)	*Aspergillus oryzae*	Submerge 40 °C for 10 weeks	Sauces	High amount of glutamate and aspartate and distinct good flavor characteristics.	Mouritsen et al. [65]
*Bombyx mori* (mulberry silkworm larvae)	*Aspergillus kawachii*	Solid state 30 °C for 3 days	Fermented powder and ethanol extract	Increased fatty acid content and reduction in free amino acid and mineral compositions. In vitro anti-cancer activity revealed characteristics cell death in human liver cancer cells.	Cho et al. [66]
*Tenebrio molitor* (yellow mealworm larvae)	Bactoferm F-LC (*Pediococcus acidilactici*, *Lactobacillus curvatus* and *Staphylococcus xylosus*)	Submerge 35 °C for 2 weeks	Fermented paste	Gave fast acidification and effectively inhibiting bacterial endospores and sulfite reducing clostridia.	Borremans et al. [63]
*Tenebrio molitor* (mealworm larvae)	*Aspergillus oryzae* and *Bacillus licheniformis*	Submerge 25 °C for 20 days	Seasoning sauces	Higher percentage nitrogen degradation rates while essential, non-essential amino acids and amino acid derivatives increased by 1.5–2.0 times.	Cho et al. [67]
*Tenebrio molitor* (mealworm larvae)	Bactoferm F-LC (*Pediococcus acidilactici*, *Lactobacillus curvatus* and *Staphylococcus xylosus*)	Submerge 35 °C for 2 weeks	Fermented paste	Mealworm paste was demonstrated to be suited for fermentation.	De Smet et al. [41]

**Table 4:** Quality attributes of fermented products containing edible insects.

Insect Species	Fermentation Type and Condition	Form of Addition	Fermented Products	Findings	References
*Acheta domesticus* (cricket)	Starter cultures 30 °C for 2 h	Powder	Composite bread	Composite bread displayed higher fatty acid, protein and essential amino acid contents. Presence of spore-forming bacteria in the substituted loaves was confirmed. 10% inclusion resulted in dough suitable for bread making and loaves with discrete overall liking.	Osimani et al. [68]
*Acheta domestica* (adult house cricket), *Hermetia illucens* (black soldier fly larvae), *Tenebrio molitor* (mealworm beetle)	Starter culture 30 °C for 90 min	Flour	Composite bread	5% insects’ flour affected dough water absorption and stability, though were suitable for bread making. Bread containing *A. domestica* presented higher amount of proteins and fibers and similar specific volume and texture indices.	González et al. [69]
*Gryllus assimilis* (cricket)	Starter culture 32 °C for 1 h	Powder	Composite-gluten free bread	Inclusion level at 10% and 20% gave gluten-free bread with acceptable reduced hardness and chewiness. 20% substitution ratio resulted in a more than twofold increase in protein and lipid content.	da Rosa Machado and Thys [70]
*Nauphoeta cinerea* (cinereous cockroach)	Starter culture 30 °C for 95 min	Flour	Composite bread	10% roasted insect flour showed satisfactory sanitary conditions. Enriched bread gave a good profile of amino acids and fatty acid contents, no negative alterations in technical characteristics and was moderately liked.	de Oliveira et al. [71]
*Schistocerca gregaria* (grasshopper)	Starter culture 1st phase: 35 °C for 45 min 2nd phase: 35 °C for 45 min	Powder	Composite bread	The inclusion level of the powder decreased the specific volume and resulted in composite breads with softer texture. 200g/kg addition showed a 60% increase in protein content.	Haber et al. [72]
*Bombyx mori* (silkworm pupae)	Starter culture 43 °C for overnight	Peptide	Yoghurt	Incorporation of the insect peptide increased acidification with a relative reduction in fermentation time. Yoghurt containing 0.5% peptide showed improved ACE inhibitory activity and amino acid content, better firmness and consistency.	Wang et al. [73]
*Tenebrio molitor* (mealworm larvae)	Natural starter (backslopping) 30 °C for 2 days	Powder	Composite flour	Successful acidification was achieved with 10 or 20% inclusion of powdered roasted mealworm larvae and thus effectively controlled the growth of Enterobacteriaceae and bacterial spores.	Klunder et al. [62]
*Tenebrio molitor* (mealworm)	Starter culture 30 °C for 1 h	Powder	Composite bread	10% mealworm bread gave the highest protein content and free amino acids while 5% addition revealed the highest specific volume and lowest firmness. Inclusion level did not negatively affect the technological features of doughs or breads, but some sensory parameters were significantly affected.	Roncolini et al. [74]
